# Association Between Metabolic Syndrome and Psychiatric Morbidity in a Nationwide Taiwanese Population Study

**DOI:** 10.3390/nu18050819

**Published:** 2026-03-03

**Authors:** Jia-In Lee, Yin-Yin Hsu, Jiun-Hung Geng, Yi-Ching Lo, Szu-Chia Chen, Cheng-Sheng Chen

**Affiliations:** 1 Graduate Institute of Medicine, College of Medicine, Kaohsiung Medical University, Kaohsiung 807, Taiwan; u9400039@gmail.com (J.-I.L.); yichlo@kmu.edu.tw (Y.-C.L.); 2 Department of Psychiatry, Kaohsiung Medical University Hospital, Kaohsiung Medical University, Kaohsiung 807, Taiwan; yinyin811121@gmail.com; 3 Department of Urology, Kaohsiung Municipal Siaogang Hospital, Kaohsiung 812, Taiwan; 4 Department of Urology, Kaohsiung Medical University Hospital, Kaohsiung Medical University, Kaohsiung 807, Taiwan; 5 Graduate Institute of Clinical Medicine, College of Medicine, Kaohsiung Medical University, Kaohsiung 807, Taiwan; 6 Research Center for Environmental Medicine, Kaohsiung Medical University, Kaohsiung 807, Taiwan; 7 Graduate Institute of Natural Products, College of Pharmacy, Kaohsiung Medical University, Kaohsiung 807, Taiwan; 8 Department of Medical Research, Kaohsiung Medical University Hospital, Kaohsiung 807, Taiwan; 9 Department of Pharmacology, School of Medicine, College of Medicine, Kaohsiung Medical University, Kaohsiung 807, Taiwan; 10 Department of Internal Medicine, Kaohsiung Municipal Siaogang Hospital, Kaohsiung Medical University, Kaohsiung 812, Taiwan; 11 Division of Nephrology, Department of Internal Medicine, Kaohsiung Medical University Hospital, Kaohsiung Medical University, Kaohsiung 807, Taiwan; 12 Faculty of Medicine, College of Medicine, Kaohsiung Medical University, Kaohsiung 807, Taiwan

**Keywords:** epidemiologic study, cross-sectional study, depression, anxiety, metabolic syndrome, risk factors, patient health questionnaire 2-item, generalized anxiety disorder 2-item

## Abstract

**Background/Objectives**: The relationship between metabolic syndrome (MetS) and mental health disorders has gained increasing attention, yet evidence from large population-based studies remains limited. This study aimed to examine the association between MetS and psychiatric morbidity in a nationwide Taiwanese adult cohort using a cross-sectional design. **Methods**: Between 2008 and 2019, a total of 121,575 adults aged 30–70 years were recruited from 29 community health screening stations across Taiwan. Demographic characteristics, lifestyle factors, medical history, and physical measurements were collected. Participants were classified as having MetS or not according to standard criteria. Psychiatric morbidity was defined as depressive and/or anxiety burden identified by validated screening instruments (Patient Health Questionnaire-2 score ≥3 or Generalized Anxiety Disorder-2 score ≥3) or self-reported physician-diagnosed depression. Multivariable logistic regression analyses were performed to evaluate the association between MetS and psychiatric morbidity after adjustment for potential confounders. **Results**: Psychiatric morbidity was identified in 1366 of 27,349 participants with MetS (5.0%) and in 4047 of 94,226 participants without MetS (4.3%). The prevalence of psychiatric morbidity was higher among participants with MetS than those without MetS (5.0% vs. 4.3%). After multivariable adjustment, MetS was significantly associated with increased odds of psychiatric morbidity (adjusted odds ratio [aOR] 1.235; 95% confidence interval [CI] 1.152–1.325). Among individual MetS components, hypertension, increased waist circumference, and hypertriglyceridemia were independently associated with higher odds of psychiatric morbidity. **Conclusions**: MetS was associated with a modest increase in psychiatric morbidity in this large Taiwanese community cohort. Because of the cross-sectional design, causal inference is limited. Future longitudinal studies are needed to clarify the direction of association and underlying mechanisms linking metabolic and mental health conditions.

## 1. Introduction

Psychiatric morbidity, particularly depression and anxiety, represents a major global public health concern, affecting millions of people worldwide [1]. According to the Global Burden of Diseases, Injuries, and Risk Factors Study (GBD) 2019, depressive and anxiety disorders rank among the leading contributors to disability related to mental illness on a global scale [2]. These disorders not only impose a profound psychological burden but are also linked to various physical comorbidities, including metabolic syndrome (MetS) [3].

MetS is characterized by a cluster of metabolic abnormalities such as central obesity, insulin resistance, dyslipidemia, and hypertension, all of which elevate the risk of cardiovascular disease and type 2 diabetes [4]. Understanding the association between psychiatric conditions and MetS has become an important research focus, as these disorders frequently coexist and may share common biological and behavioral pathways [5,6].

Recent meta-analyses have reported that individuals with depression have approximately 1.3–1.5-fold higher odds of developing MetS compared with those without depression, while MetS has also been associated with a higher prevalence of depressive and anxiety symptoms [3,5,7]. These associations may be explained by shared mechanisms, including chronic systemic inflammation [7], dysregulation of the hypothalamic–pituitary–adrenal (HPA) axis [8,9], and unhealthy lifestyle behaviors such as poor diet quality and reduced physical activity [10,11]. However, most prior studies have relied on clinical samples, relatively small cohorts, or single disease endpoints, which may limit generalizability to the general population.

Although consistent associations have been observed, the population-level burden and pattern of association remain uncertain. Community-based screening data, while not suitable for establishing causality, can provide clinically meaningful estimates of the magnitude of association in real-world populations. Furthermore, most existing studies have been conducted in Western populations, and large population-based evidence from Asian communities, particularly Taiwan, remains limited. Differences in cultural context, diet, and lifestyle may influence both metabolic risk and psychiatric expression, highlighting the need for region-specific data.

Therefore, this study used a population-based cohort in Taiwan to (1) quantify the prevalence of psychiatric morbidity according to MetS status and (2) evaluate the associations between individual MetS components, cumulative metabolic burden, and psychiatric morbidity.

## 2. Materials and Methods

### 2.1. Data Source and Study Population

**Figure 1:**
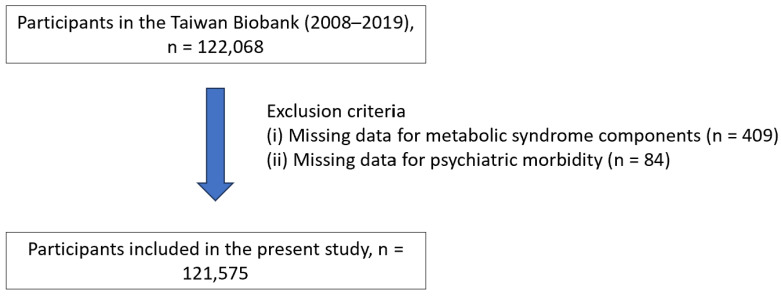
Flowchart of participant selection from the Taiwan Biobank.

The dataset for this study was derived from a nationwide, population-based cohort of volunteers aged 30–70 years who were enrolled between 2008 and 2019 through 29 community recruitment centers across Taiwan, as detailed in previous publications [12,13,14]. Participants were asked to complete standardized questionnaires that collected information on sociodemographic factors, lifestyle habits, and medical history. Mental health status was assessed using the Generalized Anxiety Disorder-2 (GAD-2) and Patient Health Questionnaire-2 (PHQ-2) instruments. Anthropometric indicators, including height, body weight, and waist circumference, were recorded, and venous blood samples were obtained for laboratory testing. Prior to participation, all individuals received a full explanation of the study’s purpose, potential benefits, and risks, and provided written informed consent. The study was conducted in accordance with the ethical principles of the Declaration of Helsinki and approved by the Institutional Review Board (IRB) of our institution (approval number: KMUHIRB-E(I)-20210058). Participants enrolled in the Taiwan Biobank between 2008 and 2019 (n = 122,068) were screened for eligibility. Individuals with missing data on metabolic syndrome components (n = 409) or psychiatric morbidity assessment (n = 84) were excluded. A total of 121,575 participants were included in the final analysis (Figure 1).

### 2.2. Variables

Study variables were obtained from the previously mentioned questionnaires and laboratory analyses. These included demographic and lifestyle factors such as age, sex, weight, height, body mass index (BMI), waist circumference, smoking behavior, alcohol consumption, physical activity, marital status, and educational level. Information on medical history and mental health status, assessed using the GAD-2 and PHQ-2 scales, was also collected. Laboratory measurements encompassed serum creatinine concentration, which was used to estimate the glomerular filtration rate (eGFR). Chronic kidney disease was defined as an eGFR below 60 mL/min/1.73 m^2^.

### 2.3. Mets Definition and Assessments

This study applied the diagnostic criteria for MetS commonly adopted in Taiwan, which are based on the recommendations of the International Diabetes Federation (IDF) and the National Cholesterol Education Program Adult Treatment Panel III (NCEP-ATP III), with adjustments to better reflect Asian population characteristics. Participants were identified as having MetS if they met at least three of the following five criteria:

(1) Abdominal obesity: Waist circumference ≥ 90 cm in men or ≥80 cm in women.
(2) Triglycerides: Fasting triglyceride levels ≥ 150 mg/dL (1.7 mmol/L) or current use of lipid-lowering medication.
(3) High-density lipoprotein cholesterol (HDL-C): HDL-C < 40 mg/dL (1.03 mmol/L) for men or <50 mg/dL (1.29 mmol/L) for women, or undergoing treatment aimed at raising HDL-C.
(4) Blood pressure: Systolic blood pressure ≥ 130 mmHg or diastolic blood pressure ≥ 85 mmHg, or use of antihypertensive medication.
(5) Fasting blood glucose: Fasting plasma glucose ≥ 100 mg/dL (5.6 mmol/L) or a documented diagnosis of diabetes under current treatment.

### 2.4. Psychiatric Morbidity

In this research, psychiatric morbidity was defined as depressive and/or anxiety burden identified by validated screening instruments (PHQ-2 ≥ 3 or GAD-2 ≥ 3) or self-reported physician-diagnosed depression. The PHQ-2 includes two items that assess depressive symptoms during the previous two weeks:

(1) Over the past two weeks, how often have you had little interest or pleasure in doing things?
(2) Over the past two weeks, how often have you felt down, depressed, or hopeless?
  Each question is rated on a four-point scale from 0 (not at all) to 3 (nearly every day).

The GAD-2 follows the same time frame and rating system, focusing on anxiety-related symptoms:

(1) During the past two weeks, how often have you felt nervous, anxious, or on edge?
(2) During the past two weeks, how often have you been unable to stop or control worrying?

For both instruments, total scores range from 0 to 6. A PHQ-2 score ≥ 3 indicated the presence of depressive symptoms, whereas a GAD-2 score ≥ 3 signified anxiety. Participants who met either cutoff or reported a prior clinical diagnosis of depression were classified as having psychiatric morbidity.

### 2.5. Statistical Analyses

Descriptive statistics were first performed to summarize the demographic and clinical characteristics of the study participants. Continuous variables were expressed as means with standard deviations (SD), whereas categorical variables were presented as frequencies and percentages. Participants were divided into two groups according to the presence or absence of metabolic syndrome. Between-group comparisons were conducted using independent t-tests for continuous variables and chi-square tests for categorical variables. To explore the associations between psychiatric morbidity and the study variables, including the presence of MetS, the number of MetS components, and each individual metabolic trait, binary logistic regression analyses were performed. Both univariable and multivariable models were constructed to adjust for potential confounders. A two-tailed *p*-value of <0.05 was considered statistically significant. All statistical analyses were performed using R software (version 3.6.2; R Foundation for Statistical Computing, Vienna, Austria) and SPSS (version 20.0; IBM Corp., Armonk, NY, USA).

### 2.6. Artificial Intelligence (AI) Use Statement

Generative AI tools (specifically ChatGPT, GPT-4; OpenAI, San Francisco, CA, USA) were used only for minor language editing and grammar correction. No AI tools were used to generate content, analyze data, or interpret results. The authors take full responsibility for the integrity and accuracy of the manuscript.

## 3. Results

### 3.1. Participant Profile

A total of 121,575 participants were analyzed, of whom 22.6% had Mets. The Mets group was older (53.6 ± 10.1 years vs. 48.8 ± 10.9 years, *p* < 0.001) and had a higher percentage of male participants (44% vs. 34%, *p* < 0.001) compared to the non-Mets group. Other significant differences included higher BMI (27.3 ± 3.8 kg/m^2^ vs. 23.3 ± 3.3 kg/m^2^, *p* < 0.001) and larger waist circumference (91.9 ± 9.1 cm vs. 80.8 ± 9.1 cm, *p* < 0.001) in the participants with Mets. The participants with Mets were also more likely to have a history of smoking (34% vs. 25%, *p* < 0.001) and alcohol use (12% vs. 8%, *p* < 0.001), along with higher rates of comorbidities such as coronary artery disease (3% vs. 1%, *p* < 0.001), asthma (4% vs. 3%, *p* < 0.001), and chronic kidney disease (3% vs. 1%, *p* < 0.001) (Table 1).

### 3.2. Univariable Analysis of Psychiatric Morbidity

In univariable logistic regression analysis (Table 2), the female participants had higher odds of psychiatric morbidity compared to the male participants (OR 1.683, 95% CI 1.581–1.791, *p* < 0.001). Smoking was associated with an increased odds of psychiatric morbidity (OR 1.243, 95% CI 1.172–1.319, *p* < 0.001), whereas being married showed a protective effect (OR 0.805, 95% CI 0.747–0.867, *p* < 0.001). Higher education level (above college vs. elementary) was associated with decreased odds of psychiatric morbidity (OR 0.844, 95% CI 0.748–0.953, *p* < 0.05). Comorbidities including coronary artery disease (OR 1.788, 95% CI 1.480–2.160, *p* < 0.001), asthma (OR 2.010, 95% CI 1.797–2.248, *p* < 0.001), and Mets (OR 1.167, 95% CI 1.096–1.243, *p* < 0.001) were also associated with an increased odds of psychiatric morbidity.

### 3.3. Multivariable Analysis of Psychiatric Morbidity

After adjusting for confounders that showed a significant risk for psychiatric morbidity in univariable analysis, including gender, smoking habit, marital status, education level, systolic blood pressure, diastolic blood pressure, history of coronary artery disease, history of asthma, and history of gastroesophageal reflux disease, female sex (adjusted OR [aOR] 2.208, 95% CI 2.045–2.384, *p* < 0.001), smoking (aOR 1.814, 95% CI 1.692–1.945, *p* < 0.001), and comorbidities including coronary artery disease (aOR 1.808, 95% CI 1.489–2.194, *p* < 0.001), asthma (aOR 1.789, 95% CI 1.596–2.004, *p* < 0.001), and gastroesophageal reflux disease (aOR 2.124, 95% CI 1.992–2.265, *p* < 0.001) remained significant predictors of psychiatric morbidity. MetS was independently associated with psychiatric morbidity (aOR 1.235, 95% CI 1.152–1.325, *p* < 0.001) (Table 3).

Moreover, a sensitivity analysis excluding participants classified solely by self-reported physician-diagnosed depression yielded similar results, with MetS remaining significantly associated with psychiatric morbidity defined by screening instruments (aOR 1.208, 95% CI 1.024–1.424, *p* = 0.025) (Supplementary Table S1). In sex-stratified analyses, the association between metabolic syndrome and psychiatric morbidity also remained significant in both men and women (men: aOR 1.340, 95% CI 1.183–1.518; women: aOR 1.199, 95% CI 1.101–1.305) (Supplementary Table S2).

### 3.4. Association Between the Number of Mets Components and Psychiatric Morbidity

Among the 90,104 individuals without MetS, 4.3% (4047/90,104) experienced psychiatric morbidity, whereas 5.0% (1366/26,058) of individuals with MetS had psychiatric morbidity. The aOR for psychiatric morbidity in participants with MetS was 1.235 (95% CI 1.152–1.325, *p* < 0.001) compared to those without MetS, as determined by multivariable regression. When analyzing the number of MetS traits, the participants with an increasing number of Mets traits also showed a progressively higher odds of psychiatric morbidity. Compared to those without traits, the participants with five traits had the highest adjusted odds (aOR 1.497, 95% CI 1.237–1.813, *p* < 0.001) (Table 4 and Supplementary Figure S1).

### 3.5. Associations Between Individual Components of Mets and Psychiatric Morbidity

Among individual components of Mets (Table 5 and Supplementary Figure S2), increased waist circumference (unadjusted OR 1.207, 95% CI 1.143–1.275), hypertriglyceridemia (unadjusted OR 1.087, 95% CI 1.018–1.161) and low HDL-C (unadjusted OR 1.169, 95% CI 1.100–1.242) were associated with a significantly increased risk of psychiatric morbidity. After adjustments, Model 1 showed that hypertension (aOR 1.269, 95% CI 1.162–1.385), impaired glucose tolerance (aOR 1.124, 95% CI 1.048–1.205), increased waist circumference (aOR 1.130, 95% CI 1.067–1.197) hypertriglyceridemia (aOR 1.169, 95% CI 1.091–1.251), and low HDL-C (aOR 1.105, 95% CI 1.039–1.175) were associated with an increased odds of psychiatric morbidity. In Model 2 (fully adjusted model), only hypertension (aOR 1.241, 95% CI 1.137–1.355), increased waist circumference (aOR 1.087, 95% CI 1.024–1.154), and hypertriglyceridemia (aOR 1.114, 95% CI 1.035–1.199) remained significantly associated with psychiatric morbidity, but not impaired glucose tolerance or low HDL-C.

## 4. Discussion

In this study, there were notable differences in demographics, lifestyle factors, and comorbidities between the participants with and without MetS. Older age, higher BMI, larger waist circumference, and higher rates of smoking and alcohol use among those with MetS may indicate underlying lifestyle or metabolic factors contributing to psychiatric morbidity, i.e., depression and/or anxiety in this study.

The higher odds of psychiatric morbidity observed among female participants are consistent with previous studies indicating greater vulnerability in women, likely influenced by hormonal fluctuations, psychosocial stressors, and behavioral differences [15,16,17]. Comorbid medical conditions, such as coronary artery disease, asthma, and gastroesophageal reflux disease, also emerged as significant predictors of psychiatric morbidity, underscoring the impact of physical health on mental well-being. Evidence from the UK Biobank suggests that genetic predisposition to coronary artery disease may contribute to its association with depression but not with anxiety, potentially reflecting distinct underlying mechanisms, such as inflammation-related pathways in depression and elevated blood pressure responses in anxiety [18,19,20].

The present findings further demonstrate that individuals with MetS have an increased likelihood of psychiatric morbidity, consistent with previous epidemiological and clinical evidence [21,22,23]. Sex-stratified analyses further demonstrated that metabolic syndrome was associated with psychiatric morbidity in both men and women, suggesting that the relationship is broadly consistent across sexes. Although the observed effect sizes were modest, the high population prevalence of metabolic syndrome implies that even small increases in relative risk may translate into a considerable mental health burden at the community level. Therefore, the findings are more relevant to population-level awareness and prevention strategies than to individual-level clinical prediction. This relationship may be explained by shared pathophysiological mechanisms, including chronic inflammation, hormonal dysregulation, and vascular injury [23,24,25]. Moreover, the stepwise increase in risk corresponding to a greater number of MetS components highlights the cumulative association of metabolic abnormalities with mental health outcomes. These findings support the importance of integrated metabolic and mental health prevention strategies in community settings.

Among individual MetS components, hypertension, increased waist circumference and hypertriglyceridemia remained significantly associated with psychiatric morbidity after full adjustment, corroborating findings from earlier research [26,27]. In contrast, impaired glucose tolerance, and low HDL-C did not retain statistical significance in the multivariable model, suggesting that their associations may be mediated indirectly through other metabolic or behavioral factors.

The overall pattern of associations aligns with the growing body of literature linking MetS and its components to depression and anxiety [7,28]. The observed bidirectional relationship between metabolic dysfunction and mental health disturbances has been well-documented in epidemiologic studies [29,30], and the current findings extend this evidence by quantifying the incremental risk associated with each additional MetS component. Prior research suggests that these associations may operate through interconnected biological and behavioral pathways. At the biological level, chronic low-grade inflammation, oxidative stress, autonomic imbalance, and hypothalamic–pituitary–adrenal axis dysregulation have been proposed as shared mechanisms contributing to both cardiometabolic dysfunction and mood-related symptoms [31,32,33,34]. At the behavioral level, depressive and anxiety symptoms may adversely affect diet quality, physical activity, sleep, and medication adherence, whereas cardiometabolic symptoms and weight-related stigma may increase psychological distress [35,36,37]. Within a cross-sectional framework, our findings are therefore more consistent with shared-pathway hypotheses than with a specific causal direction. From a nutritional perspective, diet quality is a plausible common upstream factor. Dietary patterns high in ultra-processed foods and added sugars are associated with cardiometabolic risk, and emerging evidence links healthier dietary patterns (e.g., higher intake of fiber, fruits/vegetables, and unsaturated fats) with a lower depressive symptom burden [38,39,40,41]. Although detailed dietary data were not available in this dataset, future Taiwanese cohort studies integrating nutritional assessment could help clarify whether diet mediates or modifies the MetS–mental health symptom relationship.

The large sample size in this study enhances both the precision and reliability of the results. Rigorous adjustment for potential confounding variables further strengthens the validity of the findings, reducing bias and supporting the robustness of the observed associations. The use of both univariable and multivariable logistic regression models provides a comprehensive assessment of independent predictors of psychiatric morbidity, offering deeper insight into the complex interconnections between metabolic and psychological health.

Several limitations should be acknowledged. First, the cross-sectional design precludes causal inference and does not allow determination of whether MetS precedes depressive/anxiety symptoms or vice versa. Second, PHQ-2 and GAD-2 are screening instruments; although commonly used and validated, they may misclassify symptom status compared with structured clinical interviews, and our estimates should not be interpreted as the prevalence of clinically diagnosed disorders [42,43,44]. Third, our outcome definition combined screening-positive symptoms with self-reported physician-diagnosed depression. This approach increases sensitivity for capturing population-level mental health burden but introduces heterogeneity, as screening tools identify current symptoms whereas self-reported diagnosis may reflect prior clinical recognition. The self-reported diagnosis could not be verified in this de-identified dataset. Therefore, the estimates should be interpreted as reflecting overall psychological symptom burden rather than a single uniform clinical entity or clinically confirmed psychiatric disorders. Fourth, residual confounding is possible because important factors such as medication use (psychotropic and metabolic agents), dietary patterns, psychosocial stress, monthly income, employment status, religion, and sleep were not available for adjustment. Finally, although effect sizes were modest, the large sample provides precise estimates that may still be relevant at the population level; prospective studies with repeated measurements and richer nutritional/behavioral data are needed to clarify temporal relationships and mechanisms.

## 5. Conclusions

This study demonstrates a significant association between MetS and psychiatric morbidity, including depression and anxiety. Participants with MetS exhibited higher odds of mental health disturbances, and the risk increased progressively with a greater number of MetS components. Among these components, hypertension, central obesity, and hypertriglyceridemia were key factors driving the association, underscoring the importance of effective management of these traits. In addition, sex, smoking status, marital status, and comorbidities such as coronary artery disease and asthma were identified as important predictors of psychiatric morbidity. These findings highlight the need for integrated prevention and treatment strategies that simultaneously address metabolic and psychological health to optimize overall patient outcomes. Future longitudinal studies are warranted to clarify causal pathways and inform targeted intervention programs.

## Figures and Tables

**Table 1:** Profiles of participants.

Characteristics	Total (N = 121,575)	No Metabolic Syndrome (N = 94,151)	Metabolic Syndrome (N = 27,424)	*p* Value
Age, yr	49.9 ± 11.0	48.8 ± 10.9	53.6 ± 10.1	<0.001
Male gender, n (%)	43,693 (36)	31,711 (34)	11,982 (44)	<0.001
Body mass index, kg/m^2^	24.2 ± 3.8	23.3 ± 3.3	27.3 ± 3.8	<0.001
Waist circumference, cm	83.3 ± 10.2	80.8 ± 9.1	91.9 ± 9.1	<0.001
Smoke, ever, n (%)	33,150 (27)	23,862 (25)	9288 (34)	<0.001
Alcohol status, ever, n (%)	10,357 (9)	7063 (8)	3294 (12)	<0.001
Regular exercise, yes, n (%)	49,294 (41)	38,113 (41)	11,181 (41)	0.196
Married, yes, n (%)	105,036 (86)	80,305 (85)	24,731 (90)	<0.001
Education status, n (%)				<0.001
≦High school	51,107 (42)	36,876 (39)	14,231 (52)	
≧College	70,468 (58)	57,275 (61)	13,193 (48)	
Systolic blood pressure, mm Hg	120.4 ± 18.7	116.7 ± 17.1	133.3 ± 18.0	<0.001
Diastolic blood pressure, mm Hg	73.8 ± 11.4	71.8 ± 10.6	80.8 ± 11.3	<0.001
Coronary artery disease, n (%)	1561 (1)	852 (1)	709 (3)	<0.001
Asthma, n (%)	4299 (4)	3233 (3)	1066 (4)	<0.001
Gastroesophageal reflux disease, n (%)	16,663 (14)	12,512 (13)	4151 (15)	<0.001
Chronic kidney disease, n (%)	1951 (2)	1010 (1)	941 (3)	<0.001
Individual component of metabolic syndrome				
Hypertension, n (%)	42,755 (35)	22,218 (24)	20,537 (75)	<0.001
Impaired glucose tolerance, n (%)	25,289 (21)	9841 (11)	15,448 (56)	<0.001
Increased waist circumference, n (%)	56,464 (46)	32,428 (34)	24,036 (88)	<0.001
Hypertriglyceridemi, n (%)	25,439 (21)	7904 (8)	17,535 (64)	<0.001
Low high-density lipoprotein, n (%)	31,088 (26)	13,155 (14)	17,933 (65)	<0.001

**Table 2:** Parameters associated with psychiatric morbidity in univariable binary logistic analysis.

Parameters	Odds Ratio (95% CI)	*p*
Age (per 1 year)	1.002 (1.000–1.005)	0.088
Female (vs. male)	1.683 (1.581–1.791)	<0.001
Body mass index (per 1 kg/m^2^)	0.996 (0.989–1.003)	0.272
Waist circumference (per 1 cm)	1.001 (0.999–1.004)	0.334
Smoke, ever (vs. never)	1.243 (1.172–1.319)	<0.001
Alcohol status, ever (vs. never)	1.042 (0.947–1.147)	0.401
Regular exercise, yes (vs. no)	0.958 (0.906–1.013)	0.135
Married, yes (vs. no)	0.805 (0.747–0.867)	<0.001
Education status, ≧College (vs. others)	0.791 (0.749–0.853)	<0.001
Systolic blood pressure (per 1 mm Hg)	0.995 (0.993–0.996)	<0.001
Diastolic blood pressure (per 1 mm Hg	0.992 (0.990–0.995)	<0.001
Coronary artery disease, yes (vs. no)	1.788 (1.480–2.160)	<0.001
Asthma, yes (vs. no)	2.010 (1.797–2.248)	<0.001
Gastroesophageal reflux disease, yes (vs. no)	2.242 (2.104–2.389)	<0.001
Chronic kidney disease, yes (vs. no)	1.162 (0.949–1.424)	0.147
Metabolic syndrome, yes (vs. no)	1.167 (1.096–1.243)	<0.001

**Table 3:** Parameters associated with psychiatric morbidity in multivariable binary logistic analysis.

Parameters	Adjusted Odds Ratio (95% CI)	*p*
Age (per 1 year)	-	-
Female (vs. male)	2.208 (2.045–2.384)	<0.001
Body mass index, kg/m^2^	-	-
Waist circumference, cm	-	-
Smoke, ever (vs. never)	1.814 (1.692–1.945)	<0.001
Alcohol status, ever (vs. never)	-	-
Regular exercise, yes (vs. no)	-	-
Married, yes (vs. no)	0.773 (0.716–0.835)	<0.001
Education status, ≧College (vs. others)	0.848 (0.800–0.898)	<0.001
Systolic blood pressure, mm Hg	0.995 (0.993–0.997)	<0.001
Diastolic blood pressure, mm Hg	1.002 (0.998–1.006)	0.368
Coronary artery disease, yes (vs. no)	1.808 (1.489–2.194)	<0.001
Asthma, yes (vs. no)	1.789 (1.596–2.004)	<0.001
Gastroesophageal reflux disease, yes (vs. no)	2.124 (1.992–2.265)	<0.001
Chronic kidney disease, yes (vs. no)	-	-
Metabolic syndrome, yes (vs. no)	1.235 (1.152–1.325)	<0.001

CI = Confidence interval. Adjusts for gender, smoking habit, marital status, education status, systolic blood pressure, diastolic blood pressure, history of coronary artery disease, history of asthma, and history of gastroesophageal reflux disease.

**Table 4:** Odds ratios for psychiatric morbidity by number of metabolic syndrome traits.

Variables	No. of Psychiatric Morbidity Cases/No. of Subjects (%)	Unadjusted Odds Ratio (95% CI)	*p*	Adjusted Odds Ratio (95% CI)	*p*
Metabolic syndrome (yes versus no)					
No	4047/90,104 (4.3)	1.000 (Reference)	-	1.000 (Reference)	-
Yes	1366/26,058 (5.0)	1.167 (1.096–1.243)	<0.001	1.235 (1.152–1.325)	<0.001
No. of metabolic syndrome traits					
0	1461/33,153 (4.2)	1.000 (Reference)	-	1.000 (Reference)	-
1	1428/32,100 (4.3)	1.009 (0.937–1.088)	0.804	1.076 (0.996–1.162)	0.063
2	1158/24,851 (4.5)	1.057 (0.977–1.144)	0.165	1.180 (1.083–1.285)	<0.001
3	807/15,918 (4.8)	1.150 (1.053–1.256)	0.002	1.313 (1.191–1.448)	<0.001
4	422/7759 (5.2)	1.234 (1.104–1.563)	<0.001	1.413 (1.250–1.598)	<0.001
5	137/2381 (5.4)	1.306 (1.091–1.563)	0.004	1.497 (1.237–1.813)	<0.001

CI = Confidence interval. Adjusts for gender, smoking habit, marital status, education status, systolic blood pressure, diastolic blood pressure, history of coronary artery disease, history of asthma, and history of gastroesophageal reflux disease.

**Table 5:** Odds ratios for psychiatric morbidity by individual component of metabolic syndrome.

Individual Component of Metabolic Syndrome	Unadjusted Odds Ratio (95% CI)	*p*	Model 1 Odds Ratio (95% CI)	*p*	Model 2 Odds Ratio (95% CI)	*p*
Hypertension	0.967 (0.913–1.024)	0.249	1.269 (1.162–1.385)	<0.001	1.241 (1.137–1.355)	<0.001
Impaired glucose tolerance	1.050 (0.983–1.122)	0.150	1.124 (1.048–1.205)	0.001	1.073 (0.999–1.152)	0.053
Increased waist circumference	1.207 (1.143–1.275)	<0.001	1.130 (1.067–1.197)	<0.001	1.087 (1.024–1.154)	<0.001
Hypertriglyceride	1.087 (1.018–1.161)	0.012	1.169 (1.091–1.251)	<0.001	1.114 (1.035–1.199)	0.004
Low high-density lipoprotein	1.169 (1.100–1.242)	<0.001	1.105 (1.039–1.175)	0.001	1.038 (0.971–1.109)	0.272

CI = Confidence interval. Model 1 adjusts for gender, smoking habit, marital status, education status, systolic blood pressure, diastolic blood pressure, history of coronary artery disease, history of asthma, and history of gastroesophageal reflux disease, model 2 adds individual components of metabolic syndrome.

## Data Availability

The data that support the findings of this study are available from Taiwan Biobank, but restrictions apply to their availability. These data were used under license for the current study and are not publicly available. However, they are available from the corresponding author, Jiun-Hung Geng (u9001090@hotmail.com), upon reasonable request and with permission from Taiwan Biobank.

## Supplementary Materials

The following supporting information can be downloaded at: https://www.mdpi.com/article/10.3390/nu18050819/s1, Figure S1: Adjusted odds ratios for psychiatric morbidity according to the number of metabolic syndrome components; Figure S2: Adjusted odds ratios for psychiatric morbidity according to individual metabolic syndrome components; Table S1: Sensitivity analysis: multivariable logistic regression after excluding participants classified solely by self-reported physician-diagnosed depression; Table S2: Sex-stratified multivariable logistic regression analyses for psychiatric morbidity.
