# Supplementary material for: Association Between Metabolic Syndrome and Psychiatric Morbidity in a Nationwide Taiwanese Population Study
Source: Nutrients. 2026 Mar 3;18(5):819. doi: 10.3390/nu18050819 (PMC12986950; doi:10.3390/nu18050819)
Supplement: Supplementary file 1 [file nutrients-18-00819-s001.zip › nutrients-4143449-supplementary.pdf]

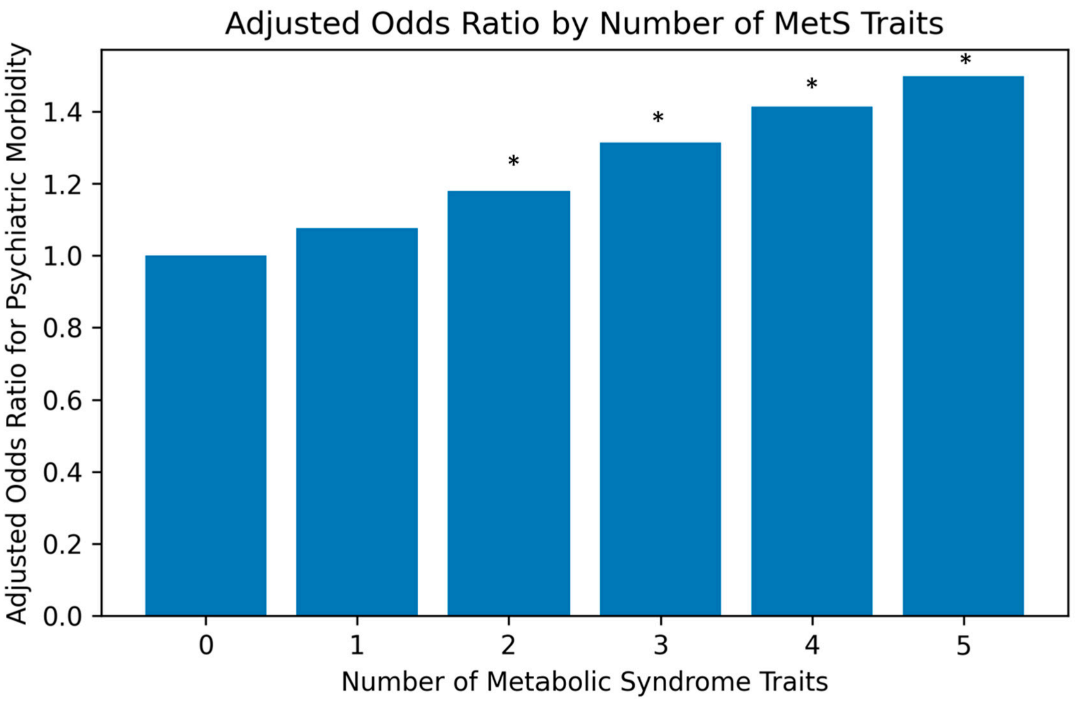

Supplementary Figure S1. Adjusted odds ratios for psychiatric morbidity according to the number of metabolic syndrome components. Bar graph illustrating the association between the number of metabolic syndrome components and psychiatric morbidity. Odds ratios were estimated using multivariable logistic regression, with 0 components as the reference group. Models were adjusted for sex, smoking status, marital status, education level, systolic blood pressure, diastolic blood pressure, history of coronary artery disease, history of asthma, and history of gastroesophageal reflux disease. * indicates $p < 0.05$.

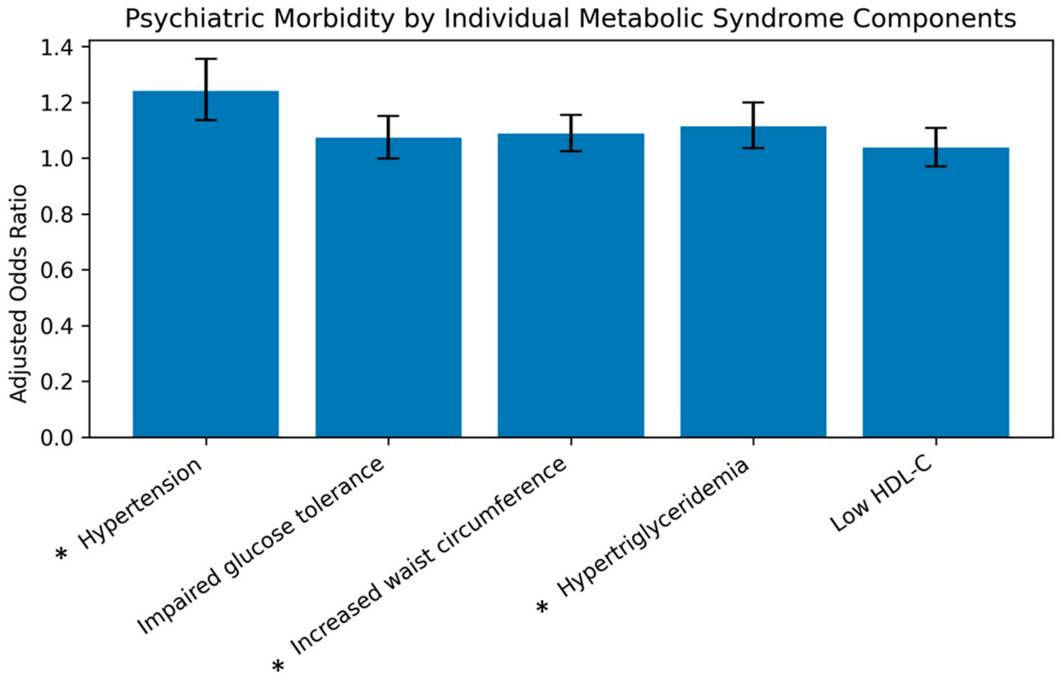

Supplementary Figure S2. Adjusted odds ratios for psychiatric morbidity according to individual metabolic syndrome components. Bars represent adjusted odds ratios from multivariable logistic regression analyses, with error bars indicating 95% confidence intervals. Odds ratios were estimated using multivariable logistic regression, with participants without any metabolic syndrome components as the reference group. Models were adjusted for sex, smoking status, marital status, education level, systolic blood pressure, diastolic blood pressure, history of coronary artery disease, history of asthma, history of gastroesophageal reflux disease, and all metabolic syndrome components simultaneously. * indicates $p < 0.05$.

Table S1. Sensitivity analysis: multivariable logistic regression after excluding participants classified solely by self-reported physician-diagnosed depression.

Variable	Adjusted Odds Ratio	95% CI	*p*
Metabolic syndrome	1.208	1.024–1.424	0.025
Female sex	1.505	1.273–1.778	<0.001
Smoking	1.668	1.420–1.959	<0.001
Ever married	0.542	0.460–0.638	<0.001
Education level	1.201	1.072–1.346	0.002
Systolic blood pressure	0.987	0.981–0.993	<0.001
Diastolic blood pressure	1.009	1.000–1.018	0.040
Asthma	1.745	1.312–2.322	<0.001
Coronary artery disease	1.674	1.018–2.754	0.042
Gastroesophageal reflux disease	1.598	1.359–1.879	<0.001

CI = Confidence interval. Adjusts for gender, smoking habit, marital status, education status, systolic blood pressure, diastolic blood pressure, history of coronary artery disease, history of asthma, and history of gastroesophageal reflux disease.

Supplementary Table S2. Sex-stratified multivariable logistic regression analyses for psychiatric morbidity.

Variable	Male<br>aOR (95% CI)	*p*	Female<br>aOR (95% CI)	*p*
Metabolic syndrome	1.340 (1.183–1.518)	<0.001	1.199 (1.101–1.305)	<0.001
Smoking experience	1.430 (1.273–1.605)	<0.001	2.155 (1.985–2.341)	<0.001
Ever married	0.506 (0.442–0.579)	<0.001	0.948 (0.862–1.041)	0.264
Education status	0.960 (0.867–1.064)	0.438	0.881 (0.835–0.930)	<0.001
Systolic blood pressure	0.993 (0.989–0.998)	0.008	0.996 (0.993–0.998)	0.002
Diastolic blood pressure	1.001 (0.994–1.009)	0.723	1.002 (0.998–1.007)	0.374
Asthma	1.530 (1.297–2.049)	<0.001	1.800 (1.578–2.053)	<0.001
Coronary artery disease	1.690 (1.277–2.237)	<0.001	2.075 (1.582–2.722)	<0.001
Gastroesophageal reflux disease	2.244 (1.977–2.547)	<0.001	2.091 (1.941–2.253)	<0.001

Adjusted for smoking experience, marital status, education status, systolic blood pressure, diastolic blood pressure, asthma, coronary artery disease, and gastroesophageal reflux disease.
aOR: adjusted odds ratio; CI: confidence interval.
